# Overview of Carbon Nanotubes for Biomedical Applications

**DOI:** 10.3390/ma12040624

**Published:** 2019-02-20

**Authors:** Juliette Simon, Emmanuel Flahaut, Muriel Golzio

**Affiliations:** 1 CIRIMAT, Université Toulouse Paul Sabatier, B.t. CIRIMAT, 118 route de Narbonne, 31062 Toulouse CEDEX 9, France; juliette.simon@chimie.ups-tlse.fr; 2 Institut de Pharmacologie et de Biologie Structurale, IPBS, Université de Toulouse Paul Sabatier, 205, Route de Narbonne, 31077 Toulouse CEDEX 4, France

**Keywords:** nanocomposites, hydrogels, diagnostic, drug delivery, tissue engineering

## Abstract

The unique combination of mechanical, optical and electrical properties offered by carbon nanotubes has fostered research for their use in many kinds of applications, including the biomedical field. However, due to persisting outstanding questions regarding their potential toxicity when considered as free particles, the research is now focusing on their immobilization on substrates for interface tuning or as biosensors, as load in nanocomposite materials where they improve both mechanical and electrical properties or even for direct use as scaffolds for tissue engineering. After a brief introduction to carbon nanotubes in general and their proposed applications in the biomedical field, this review will focus on nanocomposite materials with hydrogel-based matrices and especially their potential future use for diagnostics, tissue engineering or targeted drug delivery. The toxicity issue will also be briefly described in order to justify the safe(r)-by-design approach offered by carbon nanotubes-based hydrogels.

## 1. Introduction

**Figure 1:**
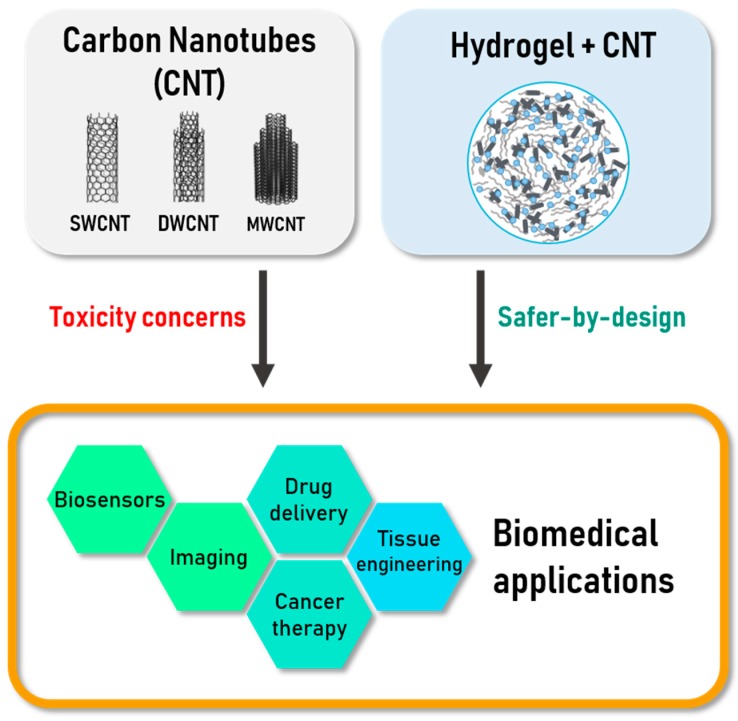
Scheme of the topics addressed in this review: Carbon nanotubes (CNTs) are good materials for various biomedical applications but they raise several question about toxicity. Their utilization as component in nanocomposites like CNTs-based hydrogels could limit those concerns.

Biomaterials are a key element of medical devices. Due to their specific properties related to the nanoscale [1], nanoparticles have progressively been introduced in biomaterials. The large ratio of surface atoms, compared to those in the bulk, increases their chemical reactivity and significantly modifies their physico-chemical properties in general (modified photocatalytic activity or even transparency for example in the case of nano TiO_2_, faster dissolution in most cases, modified electronic properties, etc.), which can be very useful in biomedical applications. If they are designed to be released, their size also allows a much faster distribution in the body. Among nanoparticles in general, carbon nanomaterials combine interesting properties such as a very high chemical resistance (no dissolution even in aggressive environments), excellent mechanical properties and a very light weight. The most used carbon nanomaterials include nanodiamonds (ND), carbon nanotubes (CNT) and graphene and its related materials (GRM: few-layer graphene (FLG), graphene oxide (GO), reduced graphene oxide (rGO)) [2]. Carbon nanomaterials also exhibit a wide range of morphologies from 0D (nanodiamonds) to nanowires (1D: carbon nanotubes) and nanosheets or nanoplatelets (2D: GRM). Among carbon nanomaterials, CNT exhibit a unique combination of mechanical, electrical and optical properties with also the possibility to fill them with different compounds including drugs [3] and are thus among the most promising nanomaterials for biomedical applications. Because of potential toxicity issues for nanomaterials in general when used as free particles, the current strategy is to favour their use in nanocomposite materials (Figure 1), as load in a biocompatible matrix (safe(r) by design approach). In this review, we have focused especially on hydrogel matrices, which are currently intensively investigated for biomedical applications.

## 2. Carbon Nanotubes (CNT) for Biomedical Applications

Carbon nanotubes are an allotropic form of carbon identified in 1991 by Iijima and since widely studied and used for a wide range of applications such as materials reinforcement, electrode materials and/or components for nanoelectronics (biosensors) or even (which could be remotely activated in some cases) drug carriers in biomedicine. They can be synthesized by different methods which will not be described in detail here but include the historical electric-arc discharge, laser ablation and the wide family of catalytic chemical vapour deposition (CCVD) techniques [4]. CNT can be described as a rolled-up graphene layer, sometimes closed at the end by fullerene caps. The number of concentric walls composing a CNT (if more than one) is an essential parameter that determines many properties. Single-wall CNT (SWCNT) have a small dimeter, most often between 1 and 2 nm, whereas multi-walled CNT (MWCNT) outer dimeter can reach ca. 100 nm. Increasing the number of layers in MWCNT inevitably also increases the number of defects and thus makes them easier to modify and to functionalise, most of the time at the cost of a degradation of their physical properties. Double-wall CNT (DWCNT) are at the interface between SWCNT and MWCNT: they exhibit many characteristics of SWCNT, such as a very narrow diameter and excellent mechanical properties but can, as MWCNT, be covalently functionalised without degrading much their electrical conductivity thanks to the presence of a second outer wall. Indeed, the question of role played by the surface chemistry of nanoparticles in general is a crucial one and CNT are no exception to the rule. It is well known that the intrinsic chemical composition and crystal structure of a nanoparticle will lead to different surface properties such as charge, hydrophobicity or hydrophilicity, possible dissolution, (photo)catalytic activity and so forth [5]. This will drive the interactions of the nanoparticle with its environment and especially the adsorption of proteins (corona). On the other hand, it has also been demonstrated that the decoration of the surface of any nanoparticle can modify their surface properties and finally lead to a rather different biological behaviour, with a marked impact on their biodistribution [6]. Similar results have also been described for CNT, which will be discussed in detail in the final section. In many cases, CNT are covalently functionalized by oxidation (HNO_3_ alone or mixed with H_2_SO_4_), leading to the grafting of oxygen-containing functional groups (hydroxyl, carboxylic acid) at the surface of the outer wall, as well as to the opening of the CNT [7]. However, in many other cases, the functionalization is non-covalent, by simple adsorption of different kinds of molecules including polymers, DNA as well as carbohydrates and derivatives [8,9,10].

Application of CNT in the biomedical field requires a few challenges to be met. The first one is related to safety and implies to use very high purity CNT in order to limit potential release of toxic ions during operation in any biological environment. This is a real challenge because high purity CNT samples usually cannot be prepared in very large scale and a compromise between quality and quantity often has to be made. The other main challenges are more related to formulation issues, not only in the biomedical field. Being able to achieve good dispersions of CNT in solvents and especially in water is one of the major ones. The strong hydrophobicity of CNT make them not only difficult to separate/individualize in a solvent but also to stabilize the suspension. This can be achieved through functionalization (covalently by chemical oxidation for example or most often non-covalently by addition of a dispersing agent or a surfactant), as has already been discussed. Another strategy, because CNT are always difficult to disperse when starting from a dry powder, is to limit or avoid drying steps during their processing. Another challenge is the typical viscosity increase related to the proper dispersion of CNT in a fluid, even at low concentration [11], which can make difficult to prepare nanocomposite materials with a high volume fraction of well-dispersed CNT.

Depending on their electronic structure, SWCNT can behave either as semiconductors or as metals. Within a random SWCNT population, there is a statistical distribution of 2/3 of semiconducting ones and 1/3 metallic of metallic ones [4]. When the number of concentric walls increases, the inter-wall interactions lead to a progressive increase in the population of metallic MWCNT. In terms of mechanical performances, they can exhibit a large surface area up to 1000 m^2^ g^−1^ in the case of DWCNT [12], a very high aspect ratio and low density [13] as well as an excellent mechanical resistance (better than steel along their axis) and an excellent flexibility when the number of walls is low (this is especially true for CCVD CNT). As described by Mohajeri et al. [14], CNT are more and more used for biomedical purposes due to their rather good biocompatibility, either for diagnostics or for the treatment of various diseases.

### 2.1. CNT Use for Diagnostic

As early diagnostic is a key point for an efficient treatment, the improvement of detection methods is especially relevant. In vitro analysis of biomarkers is already possible with good accuracy thanks to the detection immune complexes but can be time consuming and also require large amounts of biological samples when using classical dosage strategies. Due to their electronic properties, several teams have considered using CNTs as the key element of electrochemical sensors and different kind of label-free CNT-based biosensors were developed.

CNT can also act as contrast agents in different bio imaging methods [15]. Functionalised and conjugated with various biomarkers they can point out the presence and localisation of targeted cells with a rather good spatial resolution.

#### 2.1.1. Biosensors Based on CNT

In the biosensors field, CNT have been proposed as sensing element to detect and monitor several diseases, especially diabetes but also bacterial infection. For instance, Punbusayakul et al. used electrochemical monitoring of immune complexes for salmonella detection, reducing thus the detection time and facilitating the sample preparation compared to existing methods [16]. An immunosensor for adiponectin—an obesity biomarker—was also obtained by grafting oriented antibodies on DWCNT surface in order to immobilise them. A second antibody, conjugated with horse radish peroxidase (HRP)-streptavidin binds to adiponectin and reacts with the substrate during cyclic voltammetry monitoring, allowing thereby fast detection and quantification [17].

Generally speaking, the presence of CNT at the surface of the electrodes allows faster electron transfer and improves the sensitivity for electrochemical detection [18]. Field-Effect Transistor (FET)-based sensors have been reported to have excellent sensitivity [19,20], sometimes as low as attomole according to Ramnani et al. [21]. Recently resistive sensors and more precisely differential resistive pulse sensors (RPS) based on MWCNT, proved their great utility reaching the single molecule detection threshold [22].

Another recent approach demonstrated the interest of porosity as a way to immobilize molecules on biosensor electrode. Zhang et al. described a non-enzymatic glucose detector composed of porous nickel-based metal oxide framework (Ni-MOF), where the electrical conductivity was enhanced by addition of CNT. These electrodes exhibit a high glucose selectivity and this method is proposed as a relevant alternative to detection through immune complexes immobilisation [23].

#### 2.1.2. Imaging Methods Based on CNT

**Figure 2:**
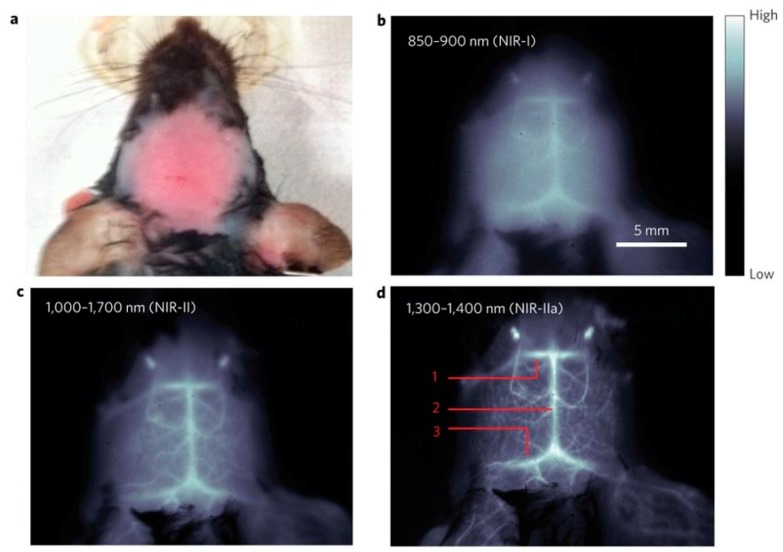
In vivo mouse brain imaging with SWNT–IRDye800 in different Near InfraRed (NIR) sub regions. (**a**) A C57Bl/6 mouse head with hair removed. (**b**–**d**), Fluorescence images of the same mouse head in the NIR-I, NIR-II and NIR-IIa regions. In (**d**), the inferior cerebral vein, superior sagittal sinus and transverse sinus are labelled 1, 2 and 3, respectively. Reprinted with permission [26].

Various technologies based on CNT are available in the imaging field. For example, photoluminescent imaging takes advantage of the fluorescence of excited SWCNT in the Near InfraRed-I (700–900 nm) and NIR-II (1100–1400 nm) ranges, wavelengths at which tissues and water are almost transparent, making possible to reach deeper penetration depths. Welsher et al. used this property to optically track in real time the biodistribution of injected SCWNTs in mice deep tissues and vessels [24,25]. SWCNT fluorescence is emerging as a reliable optical imaging method for vessels observation [26,27] (Figure 2) and was more recently used as an optical alternative to the expensive positron emission tomography-computed tomography for brown fat detection [28].

The photoacoustic effect is another interesting way to use CNT NIR absorption. Photoacoustic imaging measures the ultrasounds produced by tissues expansion around heat emitting CNT exposed to NIR stimulation. De La Zerda et al. obtained improved contrast on targeted tumour cells thanks to SWCNT conjugated with Arg-Gly-Asp peptides as well as an enhanced sensitivity and detection threshold, compare to pristine SWCNT [29,30].

Raman scattering is also an imaging option, thanks to the G band exhibited by SWCNT around 1580 cm^−1^. Liu et al. demonstrated the use of the combination of peak shifting for SWCNT depending on the ^13^C/^12^C isotopic ratio with the labelling by different targeting ligands, leading to five-colour multiplexed Raman imaging [31]. Furthermore one of the major drawback of Raman imaging, the acquisition time, may have found a major improvement with the recent development of Raman hyperspectral instrument with Bragg tuneable filter, thereby increasing impressively the scan speed and scanned surface [32]. MWCNT usually exhibit a lower signal/noise ratio compared to SWCNT and DWCNT and the sensitivity of this approach thus depends on the nature of the biological matrix.

The radioprobes presented by Hong et al. used for single-photon emission computed tomography imaging (SPECT) and composed of metal halides encapsulated within CNT, illustrate another application of CNT in bioimaging [33]. Furthermore, the proposed 1,3-dipolar cycloaddition of CNT represents a promising substitute option to widespread CNT surface oxidation as it enables the functionalisation without opening the closed ends and should thus prevent the release of radionuclides and their accumulation in thyroid and stomach. This functionalisation method was also utilised to conjugate oxidised MWCNT with gadolinium, which is an element commonly used as contrast agent for magnetic resonance imaging (MRI). Gd-MWCNT exhibited enhanced MRI contrast compared to actual commercial ones [34].

### 2.2. CNT Use for Tissue Engineering

**Figure 3:**
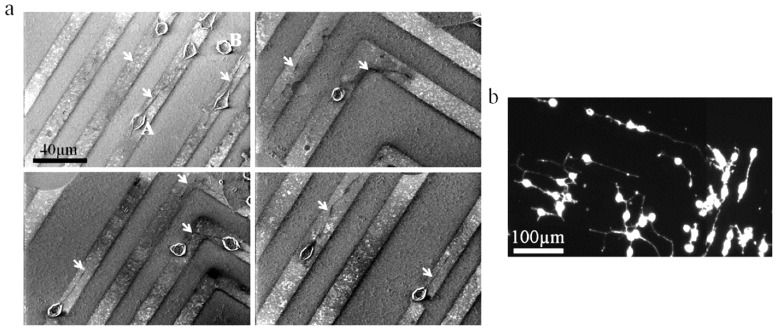
Neuro2a cells culture on a SiO_2_/CNT micropatterned surface. (**a**) SEM images of neuro2a cells cultured on patterned surfaces after 2 days of differentiation. Arrows point to neurites developed on CNT patterns. The letter A indicates a specific cell body on a CNT feature and the letter B points out a specific cell body outside of a CNT feature (on a SiO_2_ feature). (**b**) Optical fluorescence image of neural cells grown on CNT patterns after phalloidin staining. Note that neurites follow the CNT lines turning at an angle of 90°. Reprinted with permission [37].

Influence of CNT on living cells draws more and more attention as utilisation of CNT as base material for biomedical applications is increasing. Lovat et al. shown that CNT represent a good surface for cellular growth and have a promoting effect on neural signal transmission [35]. Moreover, Mazzatenta et al., by investigating the coupling model between SWCNT and hippocampal cells, have proved that SWCNT may directly stimulate brain circuit activity, highlighting SWCNT as a promising material [36]. Béduer et al. obtained similar results. They demonstrated that neurons would preferentially grow on DWCNT compared to SiO_2_ surface, due to a favourable surface texture and better adsorption of culture medium proteins, allowing patterning of neurons networks as illustrated in Figure 3. Cell differentiation was also enhanced when the growth took place on DWCNT [37]. Raw (non-functionalised) aligned MWCNT successfully sustained the growth and proliferation of pancreatic cancer cells, exhibiting a new approach for the study of this kind of cancer [38].

The ability of CNT to form 3D architecture described by Correa-Duarte et al. is equally interesting for cell proliferation enhancement and tissue engineering. The proposed MWCNT network could be shaped to fit best natural tissue morphology but also remained stable in vivo [39]. Abarrategi et al. developed CNT/chitosan meshes to support cell recolonization and observed their disassembly in vivo through dispersion in newly grown tissue [40]. Different kinds of CNT-based scaffold supporting cell colonisation were reported since [41]. The question of the potential release of CNT in case of biodegradation and the possible related toxicity issues will be discussed later on.

### 2.3. CNT Use for Targeted Therapies

Efficient drug administration is a real concern in the medical field. Indeed, low selectivity and low half-life time may lead to multiply the administrations, resulting in secondary effects and sometimes, fatal issues. Due to their expected biocompatibility and suitable size, CNT are widely studied as nanocarriers for drug and gene delivery as well as cancer treatment.

#### 2.3.1. CNT as Carrier for Drugs and Gene Delivery

The use of CNT as nanocarriers has drawn more and more attention in last years since CNT exhibit exceptional cell transfection capabilities. Liu et al. have shown that drugs such as doxorubicin (DOX) can be loaded onto the surface of poly(ethylene glycol) (PEG) conjugated SWCNT thanks to non-covalent bounds and can reach the tumorous tissue thanks to the enhanced permeability and retention (EPR) effect. A pH - release dependency would also permit delivery close to the tumour tissues [42,43]. Wells et al. relied on a mesoporous silica coating to load drugs on CNT [44]. In those examples, DOX was easily released in the acidic environment found in tumorous tissues but stayed bound to CNT at neutral and alkaline pH, limiting toxicity within the healthy body parts [42,43,44]. PEG branches also increased the hydrophilicity of SWCNT and protected the DOX bound to the nanotubes, extending its stability and lifetime in blood circulation. Another anticancer drug, 10-hydroxycamptothecin (HCPT) was successfully covalently linked to MWCNT and also exhibited the enhanced efficiency of CNT-conjugated drugs, as well as the possibility to couple imaging to this kind of treatment (theranostic approach) [45]. Recently, hydroxypropyl-β-cyclodextrin (HP-β-CD)-modified SWCNT-COOH also exhibited a rather good formononetin entrapment and a slow and sustained release [46] which might be useful for enhanced cancer treatment.

CNT have also the capacity to enter cells [47], independently of the functional groups they may have on the surface [48], allowing intracellular drugs delivery but also genes and proteins delivery. This is of particular interest for the development of gene silencing therapy, thanks to short interfering RNA (siRNA) delivery inside cytoplasm. Bartholomeusz et al. successfully dispersed SWCNT in siRNA solutions, without using any functionalisation of the nanotubes and demonstrated 90% of transfected cells within 6 h, associated with 70–80% of HIF-1 silencing and more importantly without the disadvantage of viral vectors, such as inflammation or immune response [49]. Ladeira et al. introduced siRNA in rather hard-to-transfect cells thanks to carboxyl-functionalised SWCNT with an efficiency of 96% of silencing [50]. Sanz et al. used a combination of functionalisation both outside (polyethyleneimine) and inside (chloroquine) for DNA plasmid delivery [51] In this work, the chloroquine, a lysosomotropic compound, was released from the CNT only at the low pH inside lysosomes, making possible the delivery of the DNA plasmid within the cytoplasm and finally to the cell nucleus. Gene silencing represents a concrete alternative treatment for pathologies and particularly for those localised in delicate zones such as the brain, since it has better efficiency than direct inhibitor injection [52]. The cellular uptake pathway of MWCNT β-cyclodextrin nanoplatform with branched poly(ethylenimine) (MWCNT-CD-PEI) loaded with cidofovir (Cid) was recently elucidated thanks to rhodamine (Rhod) doping and imaging by Mazzaglia et al. [53]. MWCNT-CD-PEI-Rhod were internalised through endocytosis and small drugs like Cid would escape lysosomes vesicles.

Kaboudin et al. demonstrated transfection of nucleic acid through another mechanism than endocytosis in a recent work. MWCNT functionalised with pyridine and magnetic particles transported nucleic acids bound by π-π interactions through the cell membrane and released them in the cytoplasm. A magnetic field was then applied to remove the nanocarriers from the cell, thus limiting their cytotoxicity [54]. Magnetically guided nanocarriers were also considered by Xu et al. through the one-step preparation of magnetic MWCNT using a paramagnetic surfactant coating. DNA is compacted thanks to electrostatic forces allowing the endocytosis transfection of the nanoplatform [55]. Salts shielding the interaction between the cargo and the CNT permitted the release of DNA inside the cytoplasm.

Using another strategy, Kong et al. considered the use of NIR light irradiation to help the DNA delivery. Laser stimulation of SWCNT coated by poly(ethyleneimine)-cholesterol (PEI-Chol) induced membrane permeabilization due to SWCNT photothermal activity, promoting cellular uptake and DNA release [56]. However, as remarked, further works are still needed to determine CNT pathways and fate inside tissues depending on their formulation.

#### 2.3.2. Anticancer Therapies Based on CNT

Taking advantage of CNT photothermal properties with NIR laser stimulation was considered as an elegant way for directly treating cancer. Burke et al. showed that intra-tumoral injection of MWCNT suspension, followed by short laser excitation resulted in tumour ablation in mice and enhanced survival [57]. On the other hand, intravenous injection of SWCNT conjugated with anti-CTLA-4 described by Wang et al. triggered immune reaction and enhanced cytotoxic activity additionally to the Photothermal Therapy (PTT), resulting in the destruction of the remaining nodules/metastasis [58]. The coupling of imaging methods with PTT was also proposed to promote treatment of primary tumours and detection of connected lymph nodes in a single step as illustrated [59]. Recently, MWCNT/gold nanostars hybrids have provided significant improvement in terms of photothermal conversion, making it possible to limit laser stimulation time during the therapy [60]. Furthermore, the combination of molecules delivery and PTT described by Wang et al., also used by Wells et al., broadens the application range of this cancer therapy method [44,61]. CNT-assisted PTT is however limited by laser penetration depth in tissues and still needs to prove its efficacy for thicker samples.

Most of the works presented in this section have determined the toxicity of CNT on cells during their utilisation but the fate and possible degradation of those objects were not thoroughly examined. This concern, still controversial, could be the most important drawback of CNT application in biomedicine when the nanotubes are not trapped within or at the surface of a device.

## 3. CNT-based Hydrogels for Biomedical Applications

Hydrogels are 3D polymeric cross-linked edifices where water absorption is made possible thanks to hydrophilic functional groups. Those materials can be classified in two groups depending of the nature of the cross-linking interactions. In chemical hydrogel, obtained for instance by radical polymerization or UV irradiation, the cross-linking is permanent and of covalent nature. Physical cross-linking is about electrostatic interactions and thus induces a possible reversibility aspect [62]. Hydrogels are not the only polymers used in CNT-based nanocomposites synthesis. For instance, Martinelli et al. developed cardiac tissue scaffolds composed of polydimethylsiloxane [63] and Mamidi et al. evaluated the cytotoxicity of CNT in ultrahigh molecular weight polyethylene on fibroblasts [64]. However, the following paragraphs will focus only on CNT-Hydrogel nanocomposites.

### 3.1. Biopolymer-Based Hydrogels

Hydrogels are widely used in the biomedical field due to their biocompatibility and the low inflammatory responses they induce [65]. Their porosity and hydrophilicity allow cargo molecules to be loaded, for drug delivery or for biosensing purposes [66] and their mechanical properties can be suitable for applications in tissue engineering [67]. The reversibility of physical hydrogels is also quite interesting as it can be the source of phase transitions, exploitable for in situ injection [68]. Several hydrogels can respond to different stimuli like temperature or pH variation [69], light exposure or electric field application. They represent thereby promising materials for targeted and controlled drug delivery, especially as, in some case, it can also be operated remotely.

Several polymers are suitable for hydrogel synthesis but natural polymers are more often selected for biomedical application because most are biocompatible and renewable. Some synthetic polymer also fit biocompatibility criteria (nontoxic and inducing low inflammation). Those polymers or monomers are often used combined with others, Table 1 gathers the main monomer/polymer families used for the synthesis of hydrogels. It is the reticulation of those polymers which determines the final properties of the gel. However, in spite of their potential for the various applications cited in the previous paragraph, the intrinsic properties of hydrogel matrices are limited. Their enhancement was thus investigated through the use of additional polymers or the inclusion of nanoparticles such as CNT.

### 3.2. CNT-Based Hydrogels for Diagnostic

Most biosensors developed using CNT based-hydrogel focused on glucose detection, as it is well documented. Several of them consist of electrodes allowing the monitoring of electron transfers within the redox couple glucose oxidase (GOx)-glucose. Conductive Bacterial cellulose (BC)-CNT-GOx films were directly used as electrodes by Kim et al., offering a simple and biocompatible material for cyclic voltammetry monitoring [72]. Comba et al. developed a very reliable glucose biosensor, with long-term stability using a sandwich configuration. In their work, the biosensor matrix was composed of albumin (alb), GOx and carbon nanotubes-mucin composite (CNT-muc) crosslinked with glutaraldehyde were inserted between two polycarbonate membranes [73]. Fatoni et al. used porosity to entrap the enzyme using a chitosan-bovine serum albumin (Chi-BSA) cryogel where embedded MWCNT enhanced electron transfer. The redox reaction was followed by amperometry [74].

The 3D architecture and mechanical properties of CNT-based hydrogels allow the design of different types of sensing methods. The optical glucose sensor presented by Barone et al. was based on a shift of photoluminescence (PL) emission of CNT embedded in poly(vinyl alcohol) (PVA) hydrogel, induced by apo-glucose oxidase (apoGOx) and glucose interaction. ApoGOx attached to PVA contributed to the cross-linking of the hydrogel and its interaction with glucose modified PVA conformation and hydrostatic pressure, resulting in CNT lattice deformation and change of the environment [75]. This works demonstrated the in vivo use of CNT PL with the perspective of limiting CNT release and associated toxicity concerns. Two different teams recently developed sensor-actuators reacting to glucose-GOx. In both works, hydrogel particles inserted in MWCNT twist yarn provoked swelling/de-swelling in the presence of glucose. Lee et al. took advantage of the exothermal character of the enzymatic reaction to activate the fast shrinkage of a thermally sensitive hydrogel, the poly(N-isopropylacrylamide) [76] adsorbed on MWCNT yarn. For the other team it was the binding of glucose with the boronic acid-conjugated with hyaluronic acid/cholesterol nanogel, which changed molecular interactions and swell the MWCNT yarn [77]. In the latter report, the authors observed a linear relationship between glucose concentration and the yarn’s rotation angle. A flexible and light strain sensor, suitable for human motion detection, was recently reported by Hosseini et al. Bacterial cellulose (BC)/(MWCNT) hydrogel was dried into a piezoresistive aerogel using supercritical CO_2_ drying [78].

### 3.3. CNT-Based Hydrogels Use for Tissue Engineering

**Figure 4:**
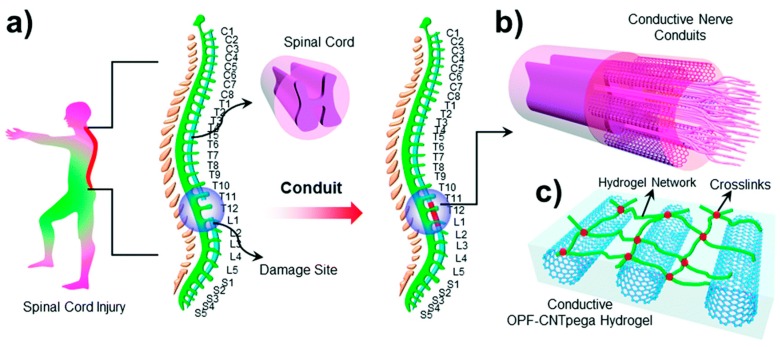
(**a**) Schematic demonstration of the spinal cord in the human body. (**b**) Conductive nerve conduits for spinal cord injury treatment. (**c**) Structure of the conductive OPF-CNTpega hydrogel. Reproduced from [88] with permission from the Centre National de la Recherche Scientifique (CNRS) and The Royal Society of Chemistry.

Maintaining the recolonization of cells in order to replace dead or harmed tissues requires scaffolding materials that respect certain properties. They should be able to form cross-linked networks, be mechanically resistant, biocompatible and should allow good cell adhesion. For such purposes, hydrogels based on biopolymers are often preferred. Furthermore, the inclusion of nanofillers such as CNT in those matrices enhance the mechanical properties [79] but also improves the electrical conductivity (when needed). For example, the regulation of the electroactive behaviour of cardiac or nervous cells during the tissue regeneration process is very important. For instance, Yildirim et al. demonstrated that the inclusion of 1 wt.% of SWCNT in an alginate scaffold was enough to improve the tensile strength close to that displayed by natural tissues, as well as increasing cell attachment and proliferation [80]. Mechanical and electrical properties of scaffolds can be tuned by modifying the CNT concentration [81] and/or orientation inside the hydrogel [82]. Ahadian et al. reported a tuneable gelatine methacyoyl (GelMA)/aligned MWCNT scaffold which enhanced the differentiation of embryoid bodies into cardiac cells, meaning this could represent a suitable support for any tissue regeneration based on stem cells therapy [82,83]. The enhancement of physical properties is particularly promising for cardiac reinforcement application, where electrical conductivity is essential but also for bone tissue regeneration. In that way, different works show that the CNT incorporation in biopolymer-based hydrogels could support the growth of cardiomyocytes fitting for cardiac tissue engineering [84] or cardiac patches [85]. Furthermore, Zhang et al. have shown the promoting effect of SWCNT on osteoblast proliferation in chitosan scaffold containing crystalline hydroxyapatite, compare to pristine hydrogels, as well as enhanced tensile and compressive modulus [86]. The scaffolds presented by Cancian et al., composed of non-covalently included CNT in thermosensitive chitosan hydrogels, exhibited appropriate viscosity for local injection, a method which could replace invasive surgeries like bone grafts [87]. Recently, Liu et al. described a promising method aiming to fill in the gap resulting of spinal cord injury (Figure 4). Nerve conduits composed of PEG functionalised CNT/oligo (poly(ethylene glycol)fumarate) (OPF) nanocomposite were generated using injection moulding technique. The described hydrogel demonstrates the enhancement of cell attachment and proliferation, confirming that the nerve conduits could help axon recovery [88].

In the field of cancer engineering, intensified cell proliferation linked to the presence of CNT can also be exploited for patterning cells growth, as shown by cancer spheroid formation in the report by Sheikholeslam et al. [89].

### 3.4. CNT-Based Hydrogels for Targeted Therapies

The use of CNT-based hydrogels for drug delivery is quite new but was investigated by many authors, as reported in the extensive review of Cirillo et al. [71]. The porous structure of biocompatible hydrogels combined with the mechanical and electrical properties improvement brought by CNT were shown to increase drug molecules stability and allow for more sustained delivery, which is of a great interest for efficient therapies [90,91]. Furthermore, this kind of material permits the development of other delivery methods, such as transdermal delivery patches.

#### 3.4.1. CNT-Based Hydrogels Use for Drug Delivery

Several teams investigated the possibility of electro-responsive CNT-based hydrogels for drug delivery. For instance, Spizzirri et al. synthesised microspheres through the polymerisation of gelatine containing MWCNT with the help of sodium methacrylate and *N*,*N*′-ethylenebisacrylamide. The drug, diclofenac sodium salt, was loaded by soaking of microspheres into a concentrated solution. The authors shown that the application of a 9V tension on the beads induced a shrinkage and thus increased the drug release [92]. This team also brought to light that the drug release behaviour was different depending on the charge of the drug. Experiments carried out on acrylamide and N,N′-ethylenebisacrylamide film polymerised by UV stimulation containing covalently introduced MWCNT, revealed that the release rate of anionic drug increased under 12V stimulation while cationic drugs were retained and released faster without electrical stimulation [93]. This was explained by the accumulation of negative charges on CNT when under 12V stimulation. Servant et al. considered the use of implantable CNT-based hydrogels for remote electrically controlled delivery. They reported methacrylic acid free-radical polymerisation in presence of pristine MWCNT to allow pulsatile drug release. The application of electrical pulses stimulated the drug release; however, they shown that the delivery rate turned back to normal quickly after the pulse end, offering more accuracy [94,95].

As for CNT-based drug carriers, pH-controlled drug delivery systems based on CNT/hydrogel nanocomposites were also considered. Peng et al. reported a different swelling of MWCNT/chitosan hydrogel depending of the pH of buffers mimicking two gastrointestinal environments, impacting the release rate [96]. Ye et al. also shown that pH had an influence on wettability and swelling of vertically aligned CNT covered by poly(methacrylic acid-co-ethylene glycol diacrylate) (P(MAA-co-EGDA)), which could lead to controlled release [97].

Wei et al. designed a dual release carrier with CNT/polycaprolactone (PCL)-PEG-PCL thermosensitive hydrogel, where DOX was loaded on CNT while Rhod was within the hydrogel. The difference of drug loading is pointed out as the source of the observed release rate. Furthermore, pH dependency was also noticed [98]. CNT based-hydrogels could also find applications in cancer therapy: Hindumathi et al. confined CNT inside a PEG cocoon in order to load drugs such as curcumin, allowing curcumin cellular uptake [99].

#### 3.4.2. CNT-Based Hydrogels Use for Skin Delivery

As Kuche et al. explained in their review work, the major drawbacks of existing transdermal delivery patches are the low drug release rate and the poor skin permeability [100]. CNT based nanocomposites could bring an innovative solution to both problems thanks to their intrinsic properties. As shown previously, this kind of hydrogel could improve drug stability through time and grant more control on the release rate. Im et al. were able to make such a patch by the electrospinning of a polyethylene oxide/pentaerythritol triacrylate/MWCNT hydrogel and they observed a correlation between drug release and applied voltage [101]. Membranes developed by Bhunia et al., made of PVA and carboxy-functionalised MWCNT [102], or by Giri et al., composed of 2-hydroxyethyl methacrylate grafted carboxymethyl guar gum and functionalised MWCNT [103], both exhibited encouraging performances for diltiazem and diclofenac release, respectively.

**Figure 5:**
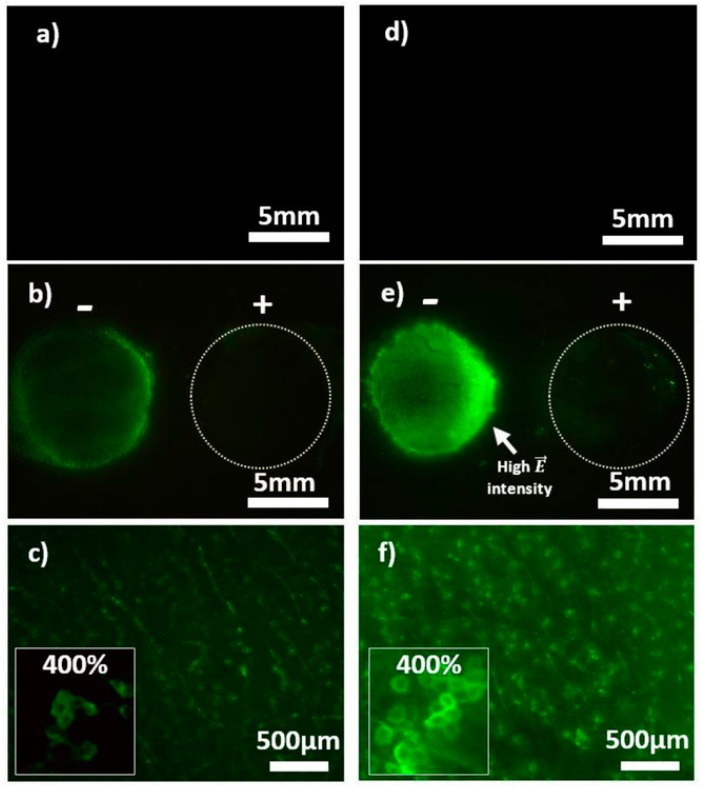
Visualization of skin electroporation. Representative pictures of mouse skin after electropermeabilisation with CTRL-AGsummuri and DWCNT-AG (1 wt. % DWCNT) containing 4 kDa FITC-dextran at 1 mm. Images (**a**,**d**) show CTRL-AG and DWCNT-AG without electrical stimulation under identical conditions at the same intensity level (×0.57). Images in (**b**,**e**) are with electrical stimulation: The anode is on the left-hand side and the cathode on the right-hand side (magnification ×0.57). (**c**,**f**) Magnifications (×4) of the anode area of (**b**,**e**), respectively. The frame on the left side of (**c**,**f**) pictures shows a numerical magnification ×400. Reprinted with permission [104].

The recent work of Guillet et al. opens a new route to solving both problems at once using CNT-based hydrogels directly as an electrode for skin electroporation. The application of high voltage electrical pulses through the skin was shown to temporarily create pores within the *stratum corneum*, which large molecules like insulin, carried by electrophoretic forces, may use to cross this natural barrier. The developed agarose/DWCNT hydrogel was use directly on mice skin as both drug storage system and electrode and demonstrated preserved mechanical properties but most importantly enhanced drug penetration into the skin, as shown in Figure 5 [104]. This work represents a promising application of CNT-based hydrogels with a very clear improvement and practical use.

Table 2 summaries the different application fields addressed in this review and the corresponding reference of cited works. In addition to this academic work, it is important to note that a few patents are also available. They all correspond to (potential) applications in the biomedical field in which CNT are included mainly to enhance the performances of existing medical devices and not for the development of new specific “nano-related” applications [105,106,107,108,109,110,111]. Furthermore, there are only few clinical trials involving CNT-based materials for biomedical applications and they mainly deal with biosensor for in vitro detection [112]. The main reason identified for this lack of practical application is most probably the toxicity issue of CNT which will be discussed in the last section.

## 4. Potential Toxicity Issues Related to CNT

The toxicity of CNT is a complex subject to address as CNT actually gather a family of nanoparticles, which properties and behaviour are related non-exhaustively to their structure, number of walls, chirality, diameter and length [113]. However, several observations can be made from the different studies which try to answer to that question. CNT may enter the organism through three different pathways: injection, ingestion and inhalation. The interferences between those nanoparticles and organs or cells exist and could be the source the varying severity and sometimes fatal issues of disease development, as described in the review work of Madani et al. [114]. Effect of several CNT on different targeted cells were also compared by Cui et al. Yet this team, as others, insists on the need of more toxicity studies on the long term in order to get more perspective on this subject [115,116].

**Figure 6:**
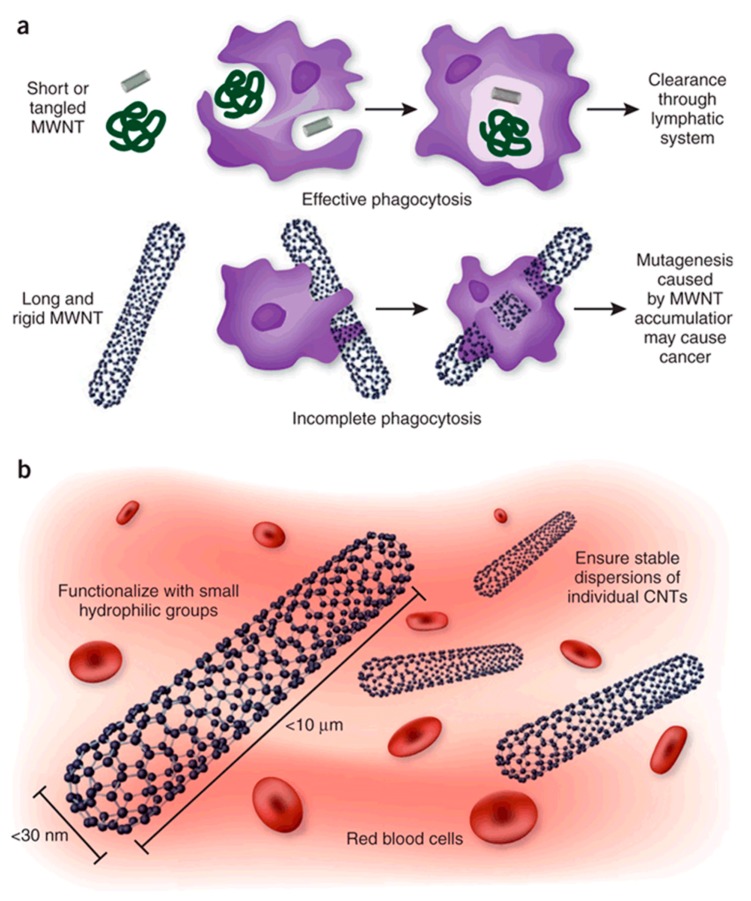
(**a**) The effect of CNT structure on phagocytosis by macrophages and clearing from tissues. Whereas macrophages can engulf MWNTs with a low aspect ratio (ratio of length to width) before their clearance by draining lymph vessels, MWNTs with a high aspect ratio cannot be cleared and accumulate in tissues, where they promote carcinogenesis. (**b**) In addition to their dimensions, other considerations relevant to the safety of CNT include increasing their solubility and preventing their aggregation, to facilitate urinary excretion and thereby prevent tissue accumulation. Reprinted with permission [123].

As described earlier in this work, CNT can transfect cells. Thanks to labelled MWCNT and further imaging it was proved that once inside the body, they could cross barriers, reach blood circulation and thus, spread within the organism [117,118]. CNT can either exit the body via urinary excretion or accumulate in secondary organs, staying indefinitely if the immune system fails to eliminate them [116]. Their biopersistence was demonstrated by Czarny and co-workers up to 12 months, without decrease [119]. CNT biodistribution is linked to several parameters such as functionalisation, dispersion and length. According to the review by Ali-Boucetta et al. on CNT toxicokinetics, covalently functionalised CNT tend to be excreted through urine whereas pristine and non-covalently functionalised CNT would accumulate in liver and spleen. CNT ability to disperse seems also a key parameter to take into consideration in toxicity. Functionalisation with small hydrophilic groups like PEG allows good and stable CNT dispersion. It is however important to know that most functionalisation processes require a former purification or activation by oxidation and that most MWCNT (in particular but not only) are significantly cut during the process, meaning in the end that it is very difficult to decorrelate the relative influence of length and functionalisation on the toxicity. The formation of CNT bundles is usually an unwanted issue as bigger agglomerate accumulate quickly, possibly leading to damages to neighbouring cells as they are easily detect by the immune system but not always phagocytosed because the length of the particles mays prevent macrophages from engulfing them properly [116,120]. Those observations are illustrated in Figure 6. The parallel is often made between CNT and asbestos as both particles are non-biodegradable fibres that the immune system fails to deal with. However, there are important differences between the two and the analogy is probably limited to the morphology and non-biodegradability as well as the activation of inflammation mechanisms when able to get within cells [121].As evidenced by some groups, CNT may however exhibit an anti-oxidant activity when they are outside the cells, by trapping of reactive oxygen species [122]. Quarantine of CNT by fibrosis could causes discomfort yet put an end to the inflammation triggered by immune system overactivity. Otherwise, the prolonged inflammation tends to degrade environment cells and lead to the development of cancer [123]. Purification step of CNT appear necessary, as the presence of metallic particles is source of oxidative stress and thus cells damaging [124,125]. Furthermore, purification method often results in CNT shortening.

A study of Allen et al. on horseradish peroxidase (HRP) degradation effect on carboxylated SWCNT suggested that polycyclic aromatic hydrocarbons (PAHs) may be found among the degradation products [126]. Moreover, carboxylated SWCNT are significantly more impacted by the HRP than pristine SWCNT which is important to consider as carboxylation is a common consequence of the purification step(s) [7]. In this sense, pristine CNT are less prone to biodegradation by peroxidases and thus more stable, while oxidized CNT biodegradation may lead to the release of toxic by-products in case of (very likely) incomplete degradation.

The use of different kinds of CNT with different morphologies and purities, with different biological models (cancer cell lines versus primary cells; in vitro versus in vivo; different animal models) at different doses using different dispersion/exposure/characterisation protocols certainly explains why the answer about the potential toxicity of CNT is still not available. The question of suitable doses for toxicity assessment is also a delicate matter. The actual exposure to CNT in real conditions is hard to quantify and concentrations used for testing the toxicity of CNT is generally far from reality. Finally the use of the weight concentration to express the exposure dose, although very practical to use, is rather irrelevant especially for comparison purposes and should be replaced by the surface concentration which is much more relevant [127].

In regard of those observations, the most cautious way to use CNT would be to prevent them from entering freely the organism. As such, trapping of CNT inside hydrogels contributes to limit the risk of direct interaction with CNT, promoting a safe-by-design development direction, while improving hydrogel properties [128]. However, to ensure no CNT release, the biocompatible polymer should be non-biodegradable and remain unaffected within the body.

## 5. Conclusions

CNT are promising materials in the biomedical field. Once functionalised to ensure their biocompatibility and conjugated with organic compounds or metallic nanoparticles, they can be used as efficient biocompatible biosensors or contrast agent for imaging but could also increase drugs lifetime within the body and facilitate their direct delivery within cells. Their usefulness in cell growth and recolonization is well illustrated, in particular for nervous cells. The use of CNT as additives inside matrices, especially biocompatible hydrogels, is an encouraging alternative way of taking advantage of their extraordinary properties while limiting the risk of direct exposure. It was shown that mechanical and electrical properties of hydrogels could be greatly improved by embedding CNT and that those nanocomposites could be used for biosensing, tissue engineering and drug delivery without the risk of CNT release into cells. Furthermore, the 3D porous structure of hydrogel widens the possible range of applications, offering more versatility, as shown by the recent development of injectable tissue scaffolds or drug delivery patches.

If the investigation of CNT local cytotoxicity is often included in recent in vivo biomedical application works and CNT generally shown not to be harmful for neighbouring cells, their fate on long term within the body is unfortunately the subject of less attention. CNT situation after they completed their intended task depends of many parameters including concentration, size and functionalisation and if most of them are generally quickly excreted, the remaining part is to stay in the body and accumulate leading to unknown long-term effects. However, as described in this work, CNT confinement inside hydrogels is a good way to limit their scattering inside the organism (as long as the hydrogel itself is not biodegradable), representing a promising way of taking advantages of those carbon nanoparticles for biomedical applications.

## Figures and Tables

**Table 1:** Bio-sourced polymers and synthetic monomers used for the synthesis of hydrogel matrices for biomedical applications [62,66,70,71].

Bio-Sourced Polymers	Synthetic Monomers
Agarose	Acrylamide (AM)
Alginate	Acrylic acid (AA)
Chitosan	Ethylene glycol (EG)
Collagen	Hydroxyethyl methacrylate (HEMA)
Dextran	Lactic acid (LA)
Fibrin	Methyl methacrylate (MMA)
Hyaluronic acid	Vinyl alcohol (VA)
Pectin	-

**Table 2:** Summary of the cited works sorted by application field.

CNT		CNT-Based Hydrogels	
Biosensors	[15,16,17,18,19,20,21,22,23]	Diagnostic	[72,73,74,75,76,77,78]
Imaging	[24,25,26,27,28,29,30,31,32,33,34]		
Cells guidance and tissue scaffolds	[35,36,37,38,39,40,41]	Cells guidance and tissue scaffolds	[79,80,81,82,83,84,85,86,87,88,89]
Drugs and gene delivery	[42,43,44,45,46,47,48,49,50,51,52,53,54,55,56]	Drugs delivery	[71,90,91,92,93,94,95,96,97,98,99]
Anticancer therapies	[44,57,58,59,60,61]	Skin delivery	[100,101,102,103,104]
